# Specific plasma metabolite profile in intestinal Behçet’s syndrome

**DOI:** 10.1186/s13023-024-03484-4

**Published:** 2025-01-13

**Authors:** Cheng-cheng Hou, Hua-fang Bao, Chun-hui She, Hua-yu Chen, Guan-xing Pan, Hua-ning Chen, Hong-bing Rui

**Affiliations:** 1 Department of Rheumatology and Immunology, The First Affiliated Hospital, Fujian Medical University, Fuzhou, 350005 Fujian China; 2 Department of Rheumatology and Immunology, National Regional Medical Center, Binhai Campus of the First Affiliated Hospital, Fujian Medical University, Fuzhou, 350212 Fujian China; 3 Department of Rheumatology and Immunology, Huadong Hospital Affiliated with Fudan University, Shanghai, China; 4 Department of Dermatology, The First Affiliated Hospital, Fujian Medical University, Fuzhou, Fujian China; 5 Fujian Dermatology and Venereology Research Institute, The First Affiliated Hospital, Fujian Medical University, Fuzhou, Fujian China; 6 Department of Pharmacy, The First Affiliated Hospital, Fujian Medical University, Fuzhou, Fujian China

**Keywords:** Behçet’s syndrome, Metabolomic analysis, Differentially abundant metabolite, Biomarkers

## Abstract

**Background:**

Intestinal Behçet’s syndrome (IBS) has high morbidity and mortality rates with serious complications. However, there are few specific biomarkers for IBS. The purposes of this study were to investigate the distinctive metabolic changes in plasma samples between IBS patients and healthy people, active IBS and inactive IBS patients, and to identify candidate metabolic biomarkers which would be useful for diagnosing and predicting IBS.

**Methods:**

In this study, we performed a global untargeted metabolomics approach in plasma samples from 30 IBS patients and 20 healthy subjects. P value < 0.05 and variable importance projection (VIP) values > 1 were considered to be statistically significant metabolites. Univariate receiver operating characteristic (ROC) curve analysis was plotted as a measure for assessing the clinical performance of metabolites, and area under curve (AUC) were assessed.

**Results:**

A total of 147 differentially abundant metabolites (DAMs) were identified between IBS patients and normal control (NC) group. The potential pathways involved in the pathogenesis of IBS include linoleic acid metabolism; GABAergic synapse; biosynthesis of unsaturated fatty acids; valine, leucine and isoleucine biosynthesis; ovarian steroidogenesis; and others. In addition, a total of 103 significant metabolites were selected to distinguish active IBS from inactive IBS patients. Tyrosine metabolism, dopaminergic synapse and neuroactive ligand-receptor interaction were found to be closely related to the disease activity of IBS. Furthermore, three potential metabolites including quinate, stearidonic acid (SDA) and capric acid (CA) could significantly differ IBS patients from NC group. On the other hand, 1-methyladenosine (m1A), genipin, methylmalonic acid (MMA) and ascorbate could significantly differentiated active IBS from inactive IBS patients.

**Conclusion:**

In conclusion, this study demonstrated the characteristic plasma metabolic profiles between IBS group and NC group, as well as between active and inactive IBS patients by using an untargeted LC/MS metabolomics profiling approach. In this study, quinate, SDA and CA were identified as potential diagnostic biomarkers for IBS. Additionally, m1A, genipin, MMA and ascorbate could serve as potential biomarkers for evaluating IBS activity. These findings might provide potential valuable insights for developing therapeutic strategies to manage IBS in the future.

**Supplementary Information:**

The online version contains supplementary material available at 10.1186/s13023-024-03484-4.

## Introduction

Behçet’s Syndrome (BS) is a variant vasculitis characterized by multisystemic inflammation, with the gastrointestinal tract is a common affected site of BS [1]. When BS patients have typical shaped ulcer in the gastrointestinal tract and clinical findings meet the diagnostic criteria for BS can be diagnosed as intestinal BS (IBS) [2]. The frequency of IBS in China seems higher compared with the Middle East and Europe. Our recent study found that intestinal involvement was the most common major organ involvement with a prevalence of 20.7%, and almost half of IBS patients (43.6%) without gastrointestinal symptoms by endoscopy [3]. However, there were few specific biomarkers of intestinal involvement in BS patients. Therefore, early detection of intestinal involvement in BS patients is extremely difficult. It is necessary to identify novel biomarkers that can be used for the diagnosis and prognostic evaluation of IBS.

Recently, clinical metabolomics have been a promising technology to provide comprehensive quantitative measurements of endogenous metabolites within biological systems. Metabolomic analysis could be used to find useful biomarkers for rheumatic disease and permit the identification of disease-specific metabolite signatures by using easily accessible biofluids [4]. Liquid chromatography mass spectrometry (LC/MS) based metabolomics is widely used in the mechanistic study and diagnosis of various diseases, such as cancer, diabetes [5]. Metabolomic analysis has been applied to investigate the unique metabolic signatures and identify biomarkers of BS in various types of biological samples [6–9]. Nonetheless, there were few metabolomic studies on the metabolic biomarkers of intestinal BS patients [9, 10].

Therefore, the purpose of this study was to investigate the distinctive metabolic changes in plasma samples between IBS patients and healthy people, as well as between active and inactive IBS, and identify candidate metabolic biomarkers for diagnosing and predicting IBS.

## Methods

### Participants and sample collection

Fifty volunteers were recruited for this study. Among them, 30 IBS patients who were treated in Huadong Hospital affiliated to Fudan University and the First Affiliated Hospital of Fujian Medical University were regarded as IBS group, and 20 age-matched healthy volunteers were regarded as normal control (NC) group. The diagnosis of IBS was confirmed with extraintestinal systemic manifestations, the characteristic endoscopic, histopathologic, and radiological features, which helped to distinguish intestinal BD from Crohn’s disease [2]. IBS patients were divided into those with active disease and inactive disease, using the simplified Behçet’s Disease Current Activity Form (BDCAF) [11]. Active disease was defined as a BDCAF value ≥ 2. Among the 30 IBS patients, 12 were active IBS patients and 18 were inactive IBS patients. The healthy volunteers included in this study did not suffer from any other autoimmune diseases and chronic diseases, including systemic lupus erythematosus, rheumatoid arthritis, hyperthyroidism, inflammatory bowel disease, hypertension, diabetes, hepatitis B and tuberculosis, etc. The demographic and clinical data including erythrocyte sedimentation rate (ESR), C-reaction protein (CRP) and BDCAF value were collected based on a standardized protocol.

Using a standard sterile procedure, blood samples were drawn from the antecubital vein after at least 10 h of fasting to minimize the effect of dietary factors. Blood samples were collected from the subjects using 5-mL EDTA anticoagulant vacuum blood collection tubes. And then, the blood samples were centrifuged at 3000 rpm for 15 min at 4 °C to separate out the supernatant-plasma, which was then frozen and stored at − 80 °C in the refrigerator until use. The study was approved by the Research Ethics Committee of the First Affiliated Hospital of Fujian Medical University (NO. [2022] 494) and written informed consent was obtained from all participants.

### Sample preparation

Untargeted metabolomics of plasma samples was performed as previously described [12], with slight modification. The plasma samples were thawed at 4 °C, and the samples were vortexed for 1 min. An aliquot of 100 µL plasma sample was transferred into the 96-well plate and precipitated with 200 µL of precooled methanol. Then, samples were vortexed for 1 min and centrifuged for 10 min at 12,000 rpm, at 4 °C. Subsequently, samples were concentrated to dryness in the A200 positive pressure nitrogen blowing module before dissolving in 150 µL of 2-chloro-l-phenylalanine solution (4 ppm) configured with 80% methanol (precooled at − 20 °C), then vortexed for 1 min and centrifuged again at 12,000 rpm and 4 °C for 10 min to obtain the supernatant for LC–MS analysis. Quality control (QC) samples were prepared by pooling the same volume of each sample to ensure instrument stability and data quality for metabolic profiling. The QC samples were evenly inserted in each set of the analysis running sequence to monitor the stability of the large-scale analysis [13].

### Metabolomics analysis by LC–MS

#### Liquid chromatography assay

The LC analysis was performed on a Vanquish UHPLC System (Thermo Fisher Scientific, USA). Chromatography was carried out with an ACQUITY UPLC® HSS T3 (2.1 × 100 mm, 1.8 µm) (Waters, Milford, MA, USA). The column maintained at 40 °C. The flow rate and injection volume were set at 0.3 mL/min and 2 μL, respectively. The liquid chromatography conditions were performed as previously described [14].

#### Mass spectrum conditions

Mass spectrometric detection of metabolites was performed on Orbitrap Exploris 120 (Thermo Fisher Scientific, USA) with ESI ion source. Simultaneous MS1 and MS/MS (Full MS-ddMS2 mode, data-dependent MS/MS) acquisition was used. The parameters were as follows: sheath gas pressure, 40 arb; aux gas flow, 10 arb; spray voltage, 3.50 kV and − 2.50 kV for ESI(+) and ESI(−), respectively; capillary temperature, 325 °C; MS1 range, m/z 100–1000; MS1 resolving power, 60,000 FWHM; number of data dependant scans per cycle, 4; MS/MS resolving power, 15,000 FWHM; normalized collision energy, 30%; dynamic exclusion time, automatic [15].

#### Data processing

The original data were firstly converted to the mzXML format in Proteowizard software package (v3.0.8789) and processed using XCMS package of R (v.3.3.2) for feature detection, retention time correction and alignment. The metabolites were identified by accuracy mass (< 30 ppm) and MS/MS data which were matched with HMDB (http://www.hmdb.ca), massbank (http://www.massbank.jp/), LipidMaps (http://www.lipidmaps.org), mzcloud (https://www.mzcloud.org) and Kyoto Encyclopedia of Genes and Genomes (KEGG) (http://www.genome.jp/kegg/), and an in-house standard database built by Panomix Biomedical Tech Co., Ltd (https://en.panomix.com, Suzhou, China). The robust LOESS signal correction (QC-RLSC) was applied for data normalization to correct for any systematic bias. After normalization, only ion peaks with relative standard deviations (RSDs) less than 30% in QC were kept to ensure proper metabolite identification. The Ropls [16] software was used for all multivariate data analyses and modelings. Data were mean-centered using scaling. Models were built on principal component analysis (PCA), orthogonal partial least-square discriminant analysis (PLS-DA) and partial least-square discriminant analysis (OPLS-DA). The metabolic profiles could be visualized as score plot, where each point represents a sample. The corresponding loading plot and S-plot were generated to provide information on the metabolites that influence clustering of the samples. All the models evaluated were tested for over fitting with methods of permutation tests. The descriptive performance of the models was determined by R2X (cumulative) (perfect model: R2X (cum) = 1) and R2Y (cumulative) (perfect model: R2Y (cum) = 1) values while their prediction performance was measured by Q2 (cumulative) (perfect model: Q2 (cum) = 1) and a permutation test. The permuted model should not be able to predict classes: R2 and Q2 values at the Y-axis intercept must be lower than those of Q2 and the R2 of the non-permuted model. OPLS-DA allowed the determination of discriminating metabolites using the variable importance in projection (VIP). The P value, VIP produced by OPLS-DA and fold change (FC) were applied to discover the contributable-variable for classification. Finally, P value < 0.05 and VIP values > 1 were considered to be statistically significant metabolites.

#### Pathway analysis

Differential metabolites were subjected to pathway analysis by MetaboAnalyst [17], which combines results from powerful pathway enrichment analysis with the pathway topology analysis. The identified metabolites in metabolomics were then mapped to the KEGG pathway for biological interpretation of higher-level systemic functions. The metabolites and corresponding pathways were visualized using KEGG Mapper tool.

#### Statistical analysis

The software of SPSS version 23.0 (SPSS Inc., Chicago, IL, USA) was used for statistical analysis. Continuous variables were expressed as mean ± standard deviation (SD). Categorical data were expressed as count and percentages. Analysis of variance (ANOVA) was used for quantitative data analysis. Non-parametric tests were applied for data with non-normal distributions. The Pearson or Spearman correlation analysis was used for evaluating the correlation between metabolites and clinical parameters of IBS patients, including ESR, CRP and BDCAF value, respectively. Univariate receiver operating characteristic (ROC) curve analysis was plotted as a measure for assessing the clinical performance of metabolites, and area under the curve (AUC) was assessed. A *P*-value < 0.05 was considered statistically significant.

## Results

### Clinical and demographic data

In this study, 30 IBS patients including 12 active and 18 inactive IBS patients and 20 healthy volunteers as NC were included. The clinical and demographic characteristics of 30 IBS patients with NC and the 12 active and 18 inactive IBS with NC were showed in Table 1. 12 active IBS patients was defined as Group IBS 1, and 18 inactive IBS patients was defined as Group IBS 2. There were no statistically significant differences in age and gender between IBS group and NC group (all *P* > 0.05). Moreover, there were also no statistically significant differences in age, gender and disease course between active and inactive IBS patients (all *P* > 0.05). However, there were statistically significant differences in ESR, CRP and BDCAF between active IBS and inactive IBS patients (all *P* < 0.05).

**Table 1:** Basic information of IBS patients and NC

Group	IBS (N = 30)	IBS 1 (n = 12)	IBS 2 (n = 18)	NC (n = 20)
*Demographics*				
Age (years, mean ± SD)	33.33 ± 11.42	35.00 ± 10.27	32.22 ± 12.28	32.70 ± 10.53
Sex (male:female)	15:15	5:7	10:8	8:12
Disease course (years, mean ± SD)	6.92 ± 6.10	9.00 ± 4.53	5.53 ± 6.69	–
ESR (mm/H, mean ± SD)	16.90 ± 19.47	32.41 ± 23.21	6.56 ± 3.91	–
CRP (mg/L, mean ± SD)	11.00 ± 23.65	23.93 ± 34.17	2.37 ± 1.49	–
BDCAF (mean ± SD)	1.10 ± 1.08	2.58 ± 0.67	0.28 ± 0.46	–
*Clinical characteristics of BS patients*				
Oral ulcer	14	12	2	–
Genital ulcer	12	12	0	–
Skin lesion	11	11	0	–
Ocular lesion	0	0	0	–
Arthritis	8	7	1	–
Gastrointestinal involvement	14	12	2	–
Epididymitis	1	1	0	–
Cardiovascular	0	0	0	–
Nervous system involvement	0	0	0	–
Blood system involvement	2	2	0	–
*Medication*				
Immunosuppressive agent	30	12	18	–
Glucocorticoid	29	12	17	–

*IBS 1* Active IBS patients; *IBS 2* Inactive IBS patients; *NC* normal contral; *SD* standard deviation; *CRP* C-reactive protein; *ESR* erythrocyte sedimentation rate; *BDCAF* Behçet’s Disease Current Activity Form

### Metabolomics analysis of plasma samples from IBS patients and NC

**Fig. 1:**
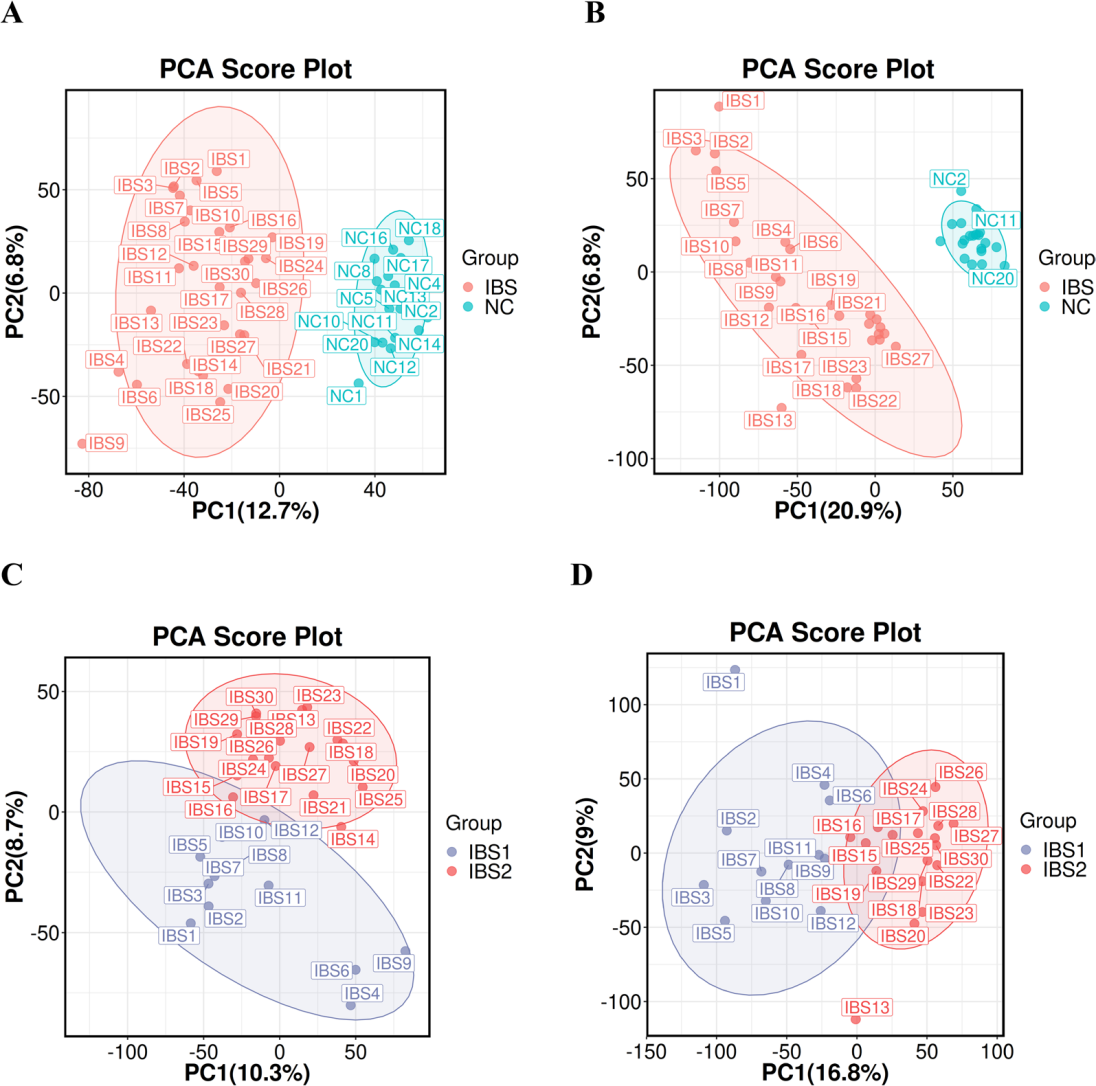
PCA models for separating IBS patients and NC. **A** PCA plot for positive ion model for separating IBS patients and NC. Pre (principal component score) = 10, for X variable dataset, model interpretability R2X = 0.456. **B** PCA plot for the negative ion model for separating IBS patients and NC. Pre (principal component score) = 8, for X variable dataset, model interpretability R2X = 0.504. **C** PCA plot for positive ion model for separating active IBS and inactive IBS patients. Pre (principal component score) = 9, for X variable dataset, model interpretability R2X = 0.519. **D** PCA plot for the negative ion model for separating active IBS and inactive IBS patients. Pre (principal component score) = 7, for X variable dataset, model interpretability R2X = 0.53. IBS 1: Active IBS patients; IBS 2: Inactive IBS patients; NC: normal contral

The typically spectra (base peak chromatogram) of positive and negative ionization modes from untargeted LC–MS metabolomics profiling analysis were shown in Fig. 1. Our results from unsupervised analyses (i.e., PCA) revealed a proper separation of all IBS patients from NC in either positive or negative ionization mode (Fig. 1A, B), suggesting a significant alteration of the metabolic profile in patients with IBS. Moreover, PCA also revealed a proper separation of active IBS patients from inactive IBS patients in either positive or negative ionization mode (Fig. 1C, D).

### Differential metabolite identification and pathway analysis from IBS patients and NC, active IBS from inactive IBS patients

**Fig. 2:**
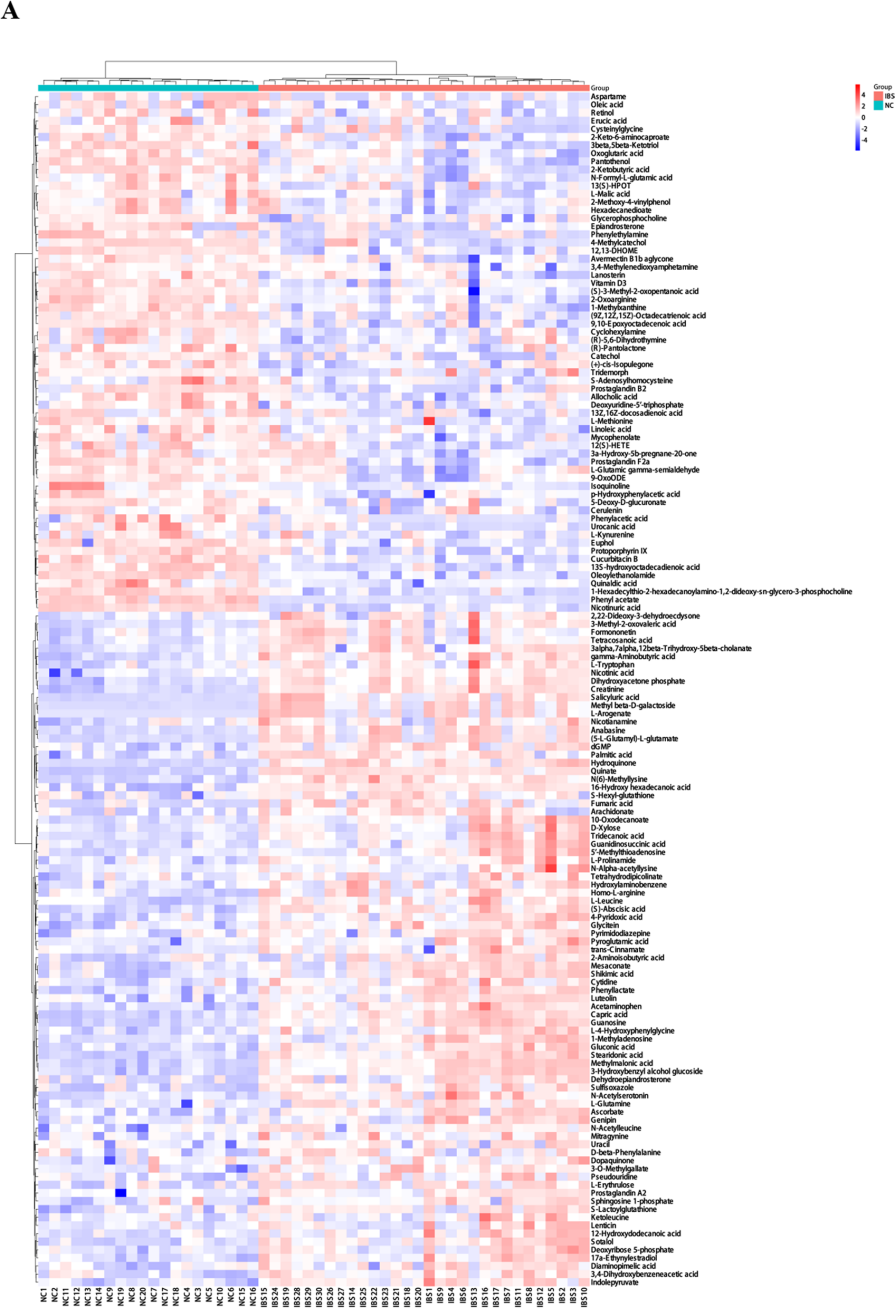

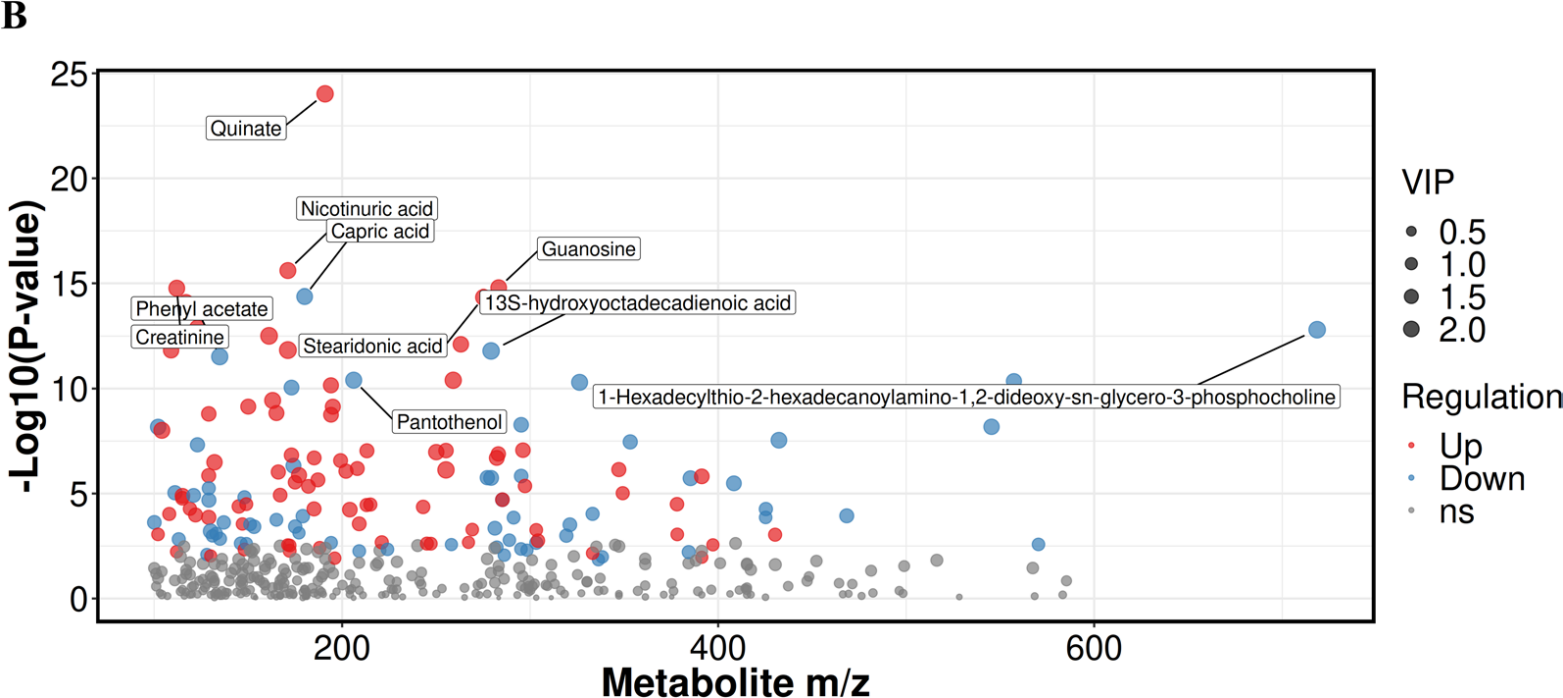

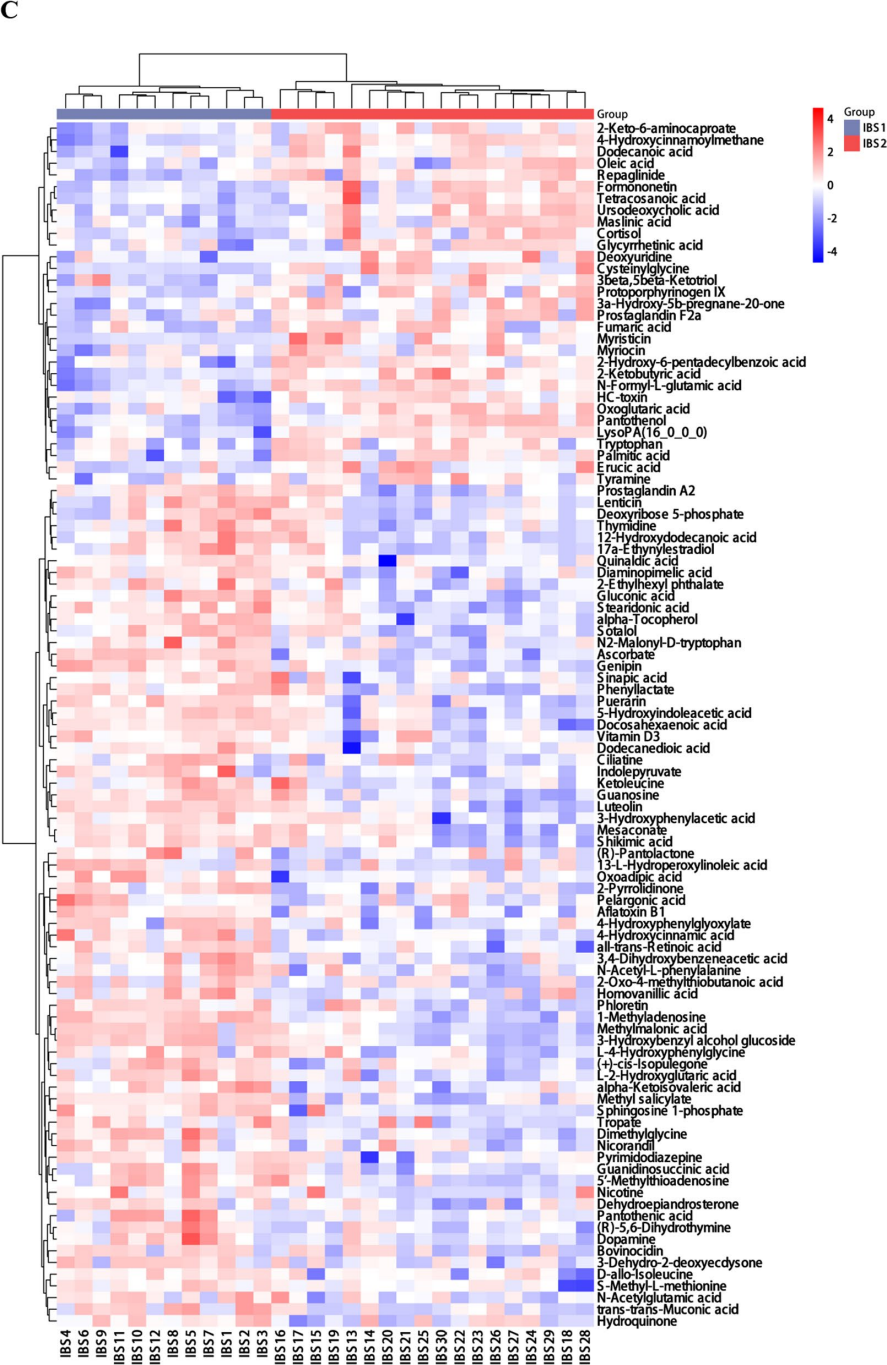

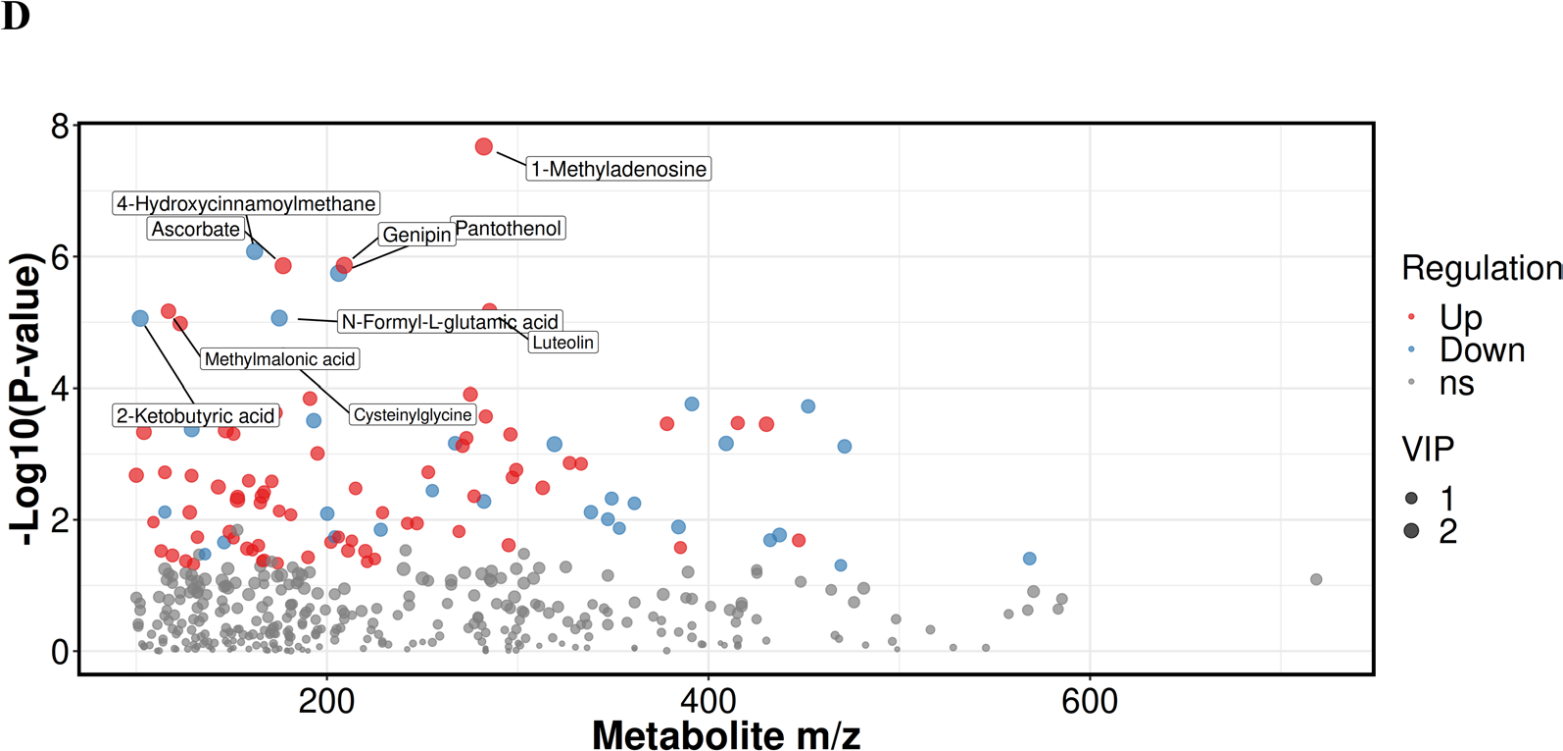
Differentially abundant metabolites between IBS patients and NC group, active IBS patients and inactive IBS patients. **A** The hierarchical clustering heat map of the 147 metabolites. The rows represent the 147 metabolites, and the columns represent samples in NC group and IBS patients. **B** Scatter plot of mass-to-charge ratio and P-value of 147 differentially abundant metabolites. **C** The hierarchical clustering heat map of the 103 metabolites. The rows represent the 103 metabolites, and the columns represent samples in active IBS and inactive IBS patients. **D** Scatter plot of mass-to-charge ratio and *P*-value of 103 differentially abundant metabolites

To reveal the plasma metabolic characteristics in IBS patients and identify and confirm high-confidence metabolites associated with IBS, we distinguished metabolites based on the criteria of VIP scores greater than 1 and *P* value less than 0.05, respectively. Thereby, a total of 147 significant metabolites were selected that can distinguish IBS patients from NC group, as shown in Supplementary Table 1. The heat map and hierarchical cluster analysis illustrate the distinct distribution patterns of the 147 potential biomarkers between the two groups. Further analysis revealed a significant increase and decrease in the relative levels of 83 and 64 differential metabolites, respectively, in patients with IBS as compared with healthy volunteers (Fig. 2A). We observed that the relative levels of quinate (VIP = 2.29; *Padj* = 9.45e−25), capric acid (VIP = 2.11; *Padj* = 2.47e−16), guanosine (VIP = 2.03; *Padj* = 1.61e−15), creatinine (VIP = 2.07; *Padj* = 1.69e−15) and stearidonic acid (VIP = 2.08; *Padj* = 4.52e−15) were markedly increased in IBS patients compared with those in NC group, whereas nicotinuric acid (VIP = 2.05; *Padj* = 4.17e−15), phenyl acetate (VIP = 2.24; *Padj* = 3.02e−12), pantothenol (VIP = 2.16; *Padj* = 3.98e−11), 13S-hydroxyoctadecadienoic acid (VIP = 2.33; *Padj* = 1.61e−12) and 1-hexadecylthio-2-hexadecanoylamino-1,2-dideoxy-sn-glycero-3-phosphocholine (VIP = 2.34; *Padj* = 1.57e−13) were prominently decreased in IBS patients (Fig. 2B).

In addition, a total of 103 significant metabolites which can distinguish active IBS from inactive IBS patients were selected, as shown in Supplementary Table 2. Further analysis revealed a significant increase and decrease in the relative levels of 72 and 31 differential metabolites, respectively, in active IBS patients as compared with inactive IBS patients (Fig. 2C). The relative levels of 1-methyladenosine (VIP = 2.97; *Padj* = 2.12e−8), genipin (VIP = 2.66; *Padj* = 1.36e−6), methylmalonic acid (VIP = 1.98; *Padj* = 6.8e−6), ascorbate (VIP = 2.58; *Padj* = 1.38e−6) and luteolin (VIP = 1.93; *Padj* = 6.66e−6) were markedly increased in active IBS patients compared with those in inactive IBS patients, whereas pantothenol (VIP = 2.71; *Padj* = 1.8e−6), 4-hydroxycinnamoylmethane (VIP = 2.70; *Padj* = 8.38e−7), 2-ketobutyric acid (VIP = 2.59; *Padj* = 8.74e−6), cysteinylglycine (VIP = 1.69; *Padj* = 3.13e−5) and N-Formyl-L-glutamic acid (VIP = 2.56; *Padj* = 8.64e−6) were prominently decreased in active IBS patients (Fig. 2D).

**Fig. 3:**
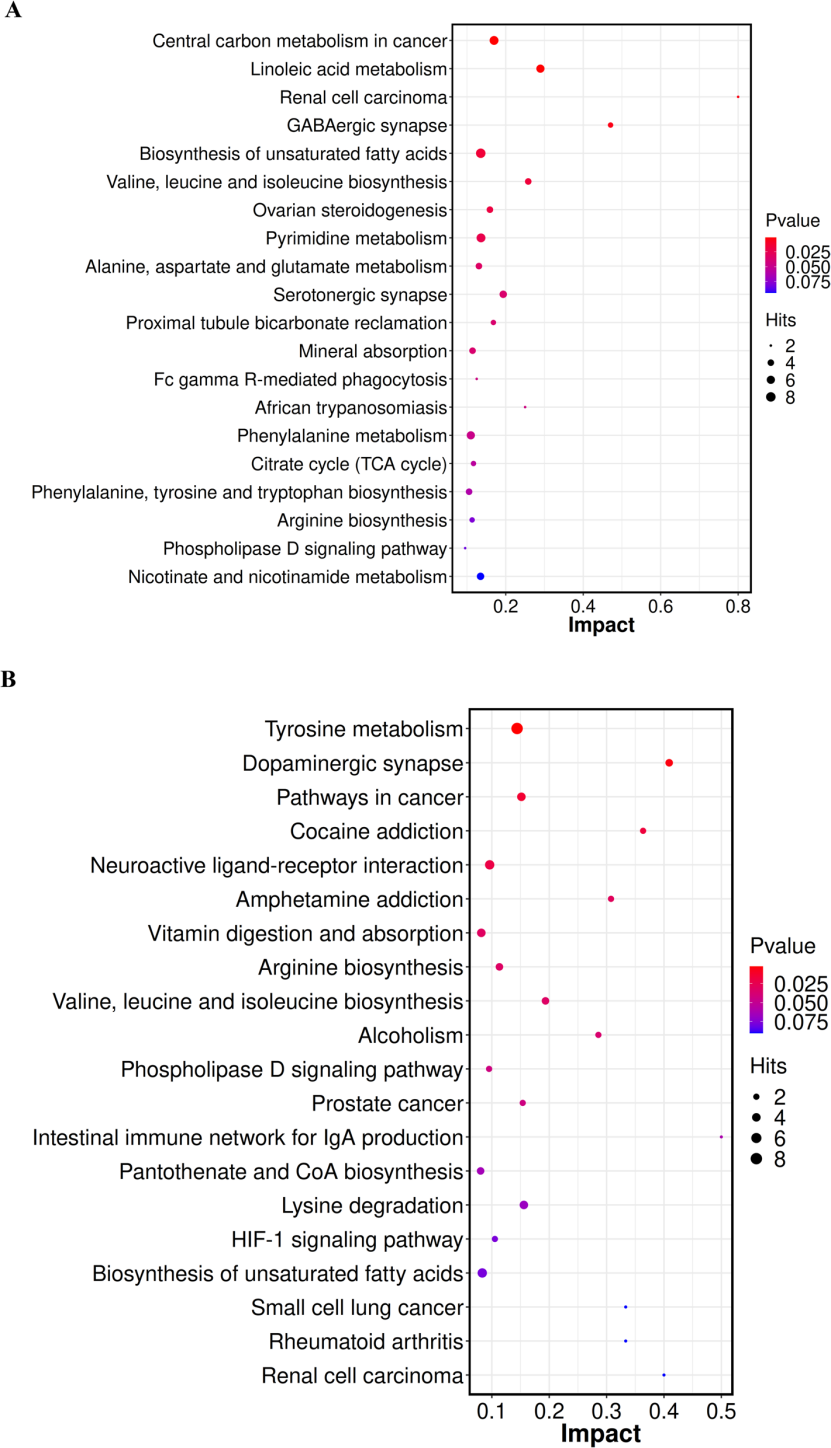
Bubble diagram of KEGG pathway analysis of differentiating metabolites enriched. **A** Bubble diagram of KEGG pathway analysis of differentiating metabolites enriched in the IBS group. **B** Bubble diagram of KEGG pathway analysis of differentiating metabolites enriched in the active IBS group. The color of the bubbles represents the value of adjusted P value, and the size of bubbles represents the number of counts (sorted by enrichment ratio)

Next, these differential metabolites were entered as input data to perform the pathway enrichment analysis. The detailed results of the pathway analysis are shown in Fig. 3. The potential pathways participating in the pathogenesis of BS include linoleic acid metabolism; GABAergic synapse; biosynthesis of unsaturated fatty acids; valine, leucine and isoleucine biosynthesis, and ovarian steroidogenesis; etc. (Fig. 3A). Furthermore, tyrosine metabolism, dopaminergic synapse and neuroactive ligand-receptor interaction were found to be closely related to the disease activity of IBS (Fig. 3B). These results provide biological relevance between IBS patients and control subjects, as well as between active and inactive IBS patients.

Finally, we also analyzed the differential metabolites and conducted KEGG pathway analysis among the active IBS, inactive IBS and NC group (Supplementary Fig. 2).

### Selection of potential diagnostic biomarkers of IBS

**Fig. 4:**
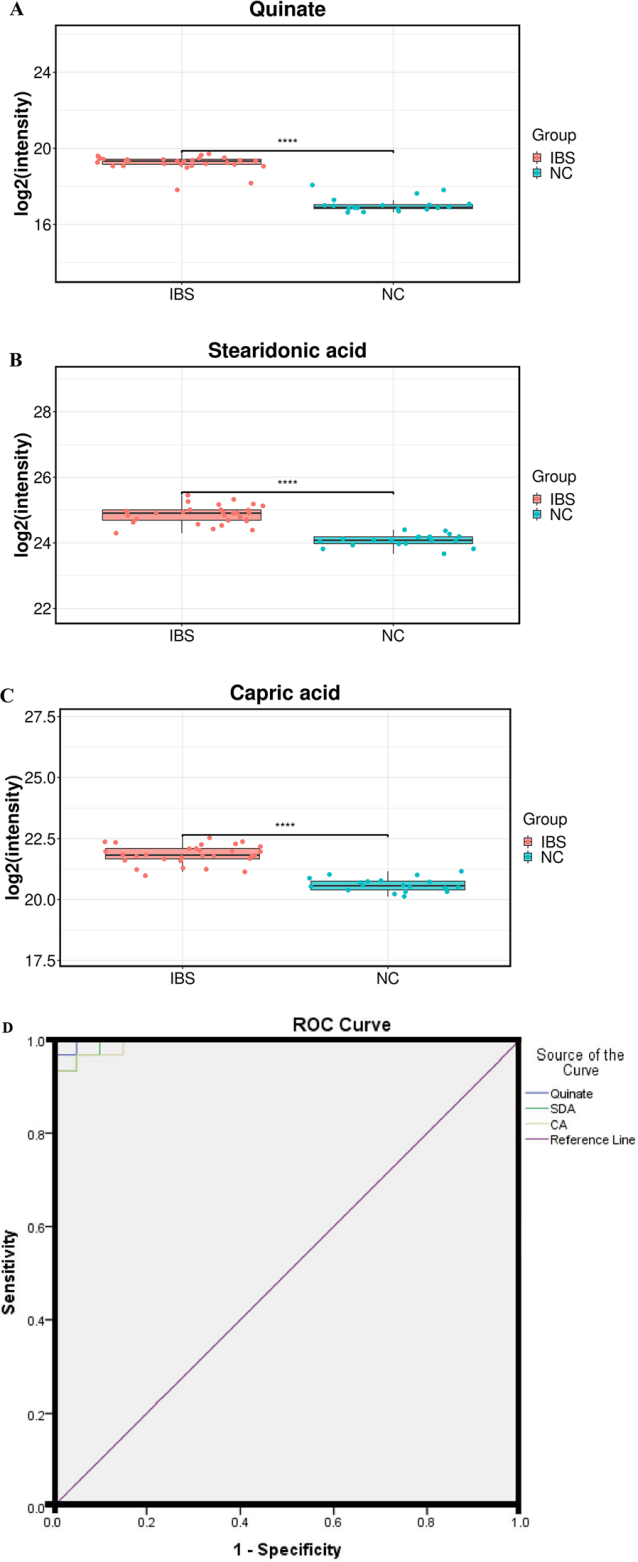
Box plots of metabolites and Receiver operating characteristic (ROC) curve model to discriminate IBS from NC group. **A** Quinate; **B** SDA; **C** CA; **D** ROC curve model of metabolites to discriminate IBS patients from NC group. SDA: stearidonic acid; CA: capric acid; NC: normal contral

To evaluate the clinical diagnostic ability of the metabolite candidates for IBS and to assess the disease activity of IBS, box plots and classical univariate ROC curve analyses were applied. Statistically significant differences were observed in quinate, stearidonic acid (SDA) and capric acid (CA) between IBS patients and NC group (Fig. 4A–C, all P < 0.001). Among them, the ROC curves of quinate, SDA and CA showed a relatively higher diagnostic performance for distinguishing IBS from the NC group with AUC values of 0.998 (95% CI 0.993–1.000), 0.995 (95% CI 0.984–1.000) and 0.993 (95% CI 0.979–1.000) (Fig. 4D).

**Fig. 5:**
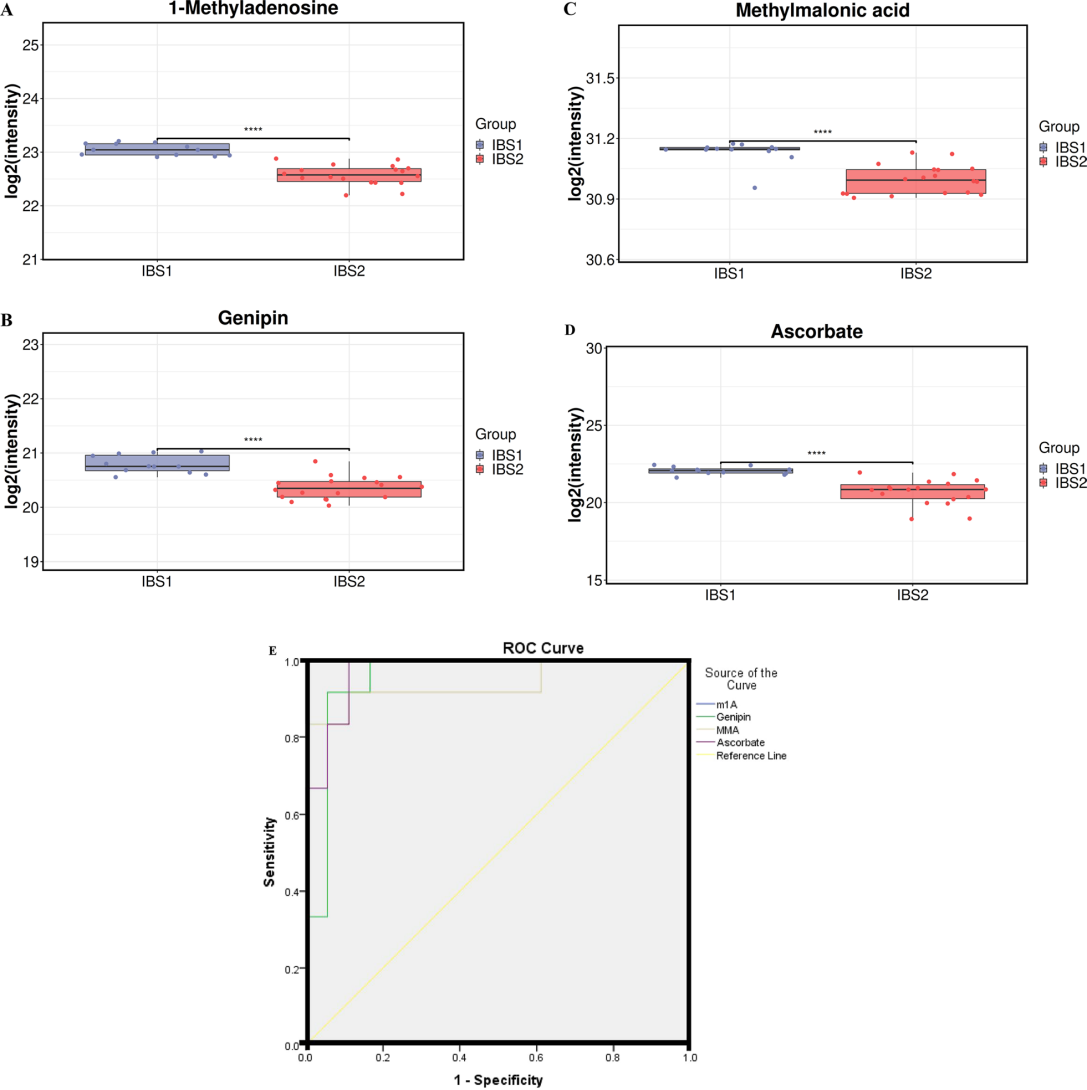
Box plots of metabolites and ROC curve model to discriminate the active IBS from inactive IBS. **A** m1A; **B** Genipin; **C** MMA; **D** Ascorbate; **E** ROC curve model of metabolites to discriminate active IBS from inactive IBS. m1A: 1-methyladenosine; MMA: methylmalonic acid; IBS 1: Active IBS patients; IBS 2: Inactive IBS patients

On the other hand, there were significant differences in 1-methyladenosine (m1A), genipin, methylmalonic acid (MMA) and ascorbate between the active IBS and the inactive IBS patients (Fig. 5A–D, all *P* < 0.001). Furthermore, ROC curves were used to show the sensitivity and specificity of the above metabolites to predict the activity of IBS. The AUC of m1A, genipin, MMA and ascorbate for predicting IBS disease activity was 1.00 (95% CI 1.000–1.000), 0.954 (95% CI 0.877–1.000), 0.940 (95% CI 0.839–1.000) and 0.972 (95% CI 0.923–1.000), respectively (Fig. 5E). Finally, the correlation between the expression level of the above metabolites and the index of BS disease activity was analyzed. The results suggested that m1A, genipin, MMA and ascorbate were positively correlated with ESR, CRP and BDCAF (all *P* < 0.05, Table 2).

**Table 2:** Correlation between differentially abundant metabolites and clinical indicators in IBS patients

Variables	ESR	CRP	BDCAF
*r* (m1A)	0.616	0.453	0.766
*P* (m1A)	0.000	0.012	0.000
*r* (Genipin)	0.631	0.514	0.617
*P* (Genipin)	0.000	0.000	0.000
*r* (MMA)	0.578	0.384	0.674
*P* (MMA)	0.001	0.036	0.000
*r* (Ascorbate)	0.546	0.391	0.790
*P* (Ascorbate)	0.002	0.033	0.000

*m1A* 1-methyladenosine; *MMA* methylmalonic acid; *CRP* C-reactive protein; *ESR* erythrocyte sedimentation rate; *BDCAF* Behçet’s Disease Current Activity Form

## Discussion

The diagnosis of BS has been a major challenge for rheumatologists due to its lack of specific clinical diagnostic biomarkers. IBS is a common clinical subtype of BS, and IBS patients tend to be more insidious and exhibit fewer clinical symptoms. Metabolomics is a promising technology for clinical applications, including biomarkers for the diagnosis and prognosis of diseases. Although there have been some studies that have applied metabolomics to identify biomarkers of BS in serum, plasma, urine and sweat [6–9, 17], no research has focused on the application of metabolomics for diagnosing IBS and identifying disease activity biomarkers.

Given that different subtypes of BS show diverse metabolic landscapes, we performed metabolic profiling to identify potential plasma metabolic biomarkers in IBS patients. In the present study, we demonstrated the characteristic plasma metabolic profiles between IBS group and the NC group, as well as between active and inactive IBS. As a result, 147 significant metabolites including 83 increased metabolites and 64 decreased metabolites were selected to distinguish IBS patients from the NC group (Fig. 2A). For instance, the relative levels of quinate, capric acid, guanosine, creatinine and stearidonic acid were significantly increased, whereas nicotinuric acid, phenyl acetate, pantothenol, 13S-hydroxyoctadecadienoic acid and 1-hexadecylthio-2-hexadecanoylamino-1,2-dideoxy-sn-glycero-3-phosphocholine were prominently decreased in IBS patients compared with those in the NC group (Fig. 2B). Additionally, 103 significant metabolites including 72 increased and 31 decreased metabolites were selected to distinguish active IBS from inactive IBS patients (Fig. 2C). This study has shown that the relative levels of 1-methyladenosine, genipin, luteolin, ascorbate and methylmalonic acid were markedly increased, whereas those of pantothenol, 4-hydroxycinnamoylmethane, 2-ketobutyric acid, cysteinylglycine and N-formyl-L-glutamic acid were prominently decreased in active IBS patients (Fig. 2D). Ma et al. [18] reported a panel consisting of 5 serum metabolites could diagnose Crohn’s disease and distinguish it from IBS, which were not among the differentially abundant metabolites listed above. In our study, the enrichment analysis indicated that the differentially abundant metabolites between IBS patients and NC were highly enriched in multiple signaling pathways, including linoleic acid metabolism, GABAergic synapse and biosynthesis of unsaturated fatty acids. Furthermore, tyrosine metabolism, dopaminergic synapse, cocaine addiction and neuroactive ligand-receptor interaction were found to be closely related to the disease activity of IBS. Zheng WJ [17] and Ahn JK et al. [6] found linoleic acid was potential metabolite biomarker for diagnosis of BS, which suggested linoleic acid metabolism may play an important role in the pathogenesis of BS. GABAergic synapse has been reported to be connected to gastrointestinal stress-related disorders including inflammatory bowel disease [19]. Additionally, a previous study [17] also found that the biosynthesis of unsaturated fatty acids was enriched and involved in the pathogenesis of BS, which was consistent with our results. Chen S et al. [20] reported that tyrosine phosphatase activity and immunoglobulin binding were enriched in BS. Furthermore, tyrosine kinase receptor signaling was also reported to be contribute to the development of BS [21], which suggested tyrosine metabolism may be extremely important during the course of BS. Dopaminergic synapse, cocaine addiction and neuroactive ligand-receptor interaction have not previously been reported in BS. The gut-brain axis is a bilateral communication network between the gastrointestinal tract and the central nervous system [22]. Compelling evidence has indicated that neurotransmitters including catecholamines, serotonin, dopamine have an important physiological role in various digestive diseases [23]. Since dopaminergic synapse, cocaine addiction and neuroactive ligand-receptor interaction pathways were discovered in IBS patients, they may play an important role through the gut-brain axis in intestinal involvement in BS.

Finally, three potential metabolites quinate, SDA and CA were found to be potential biomarkers for diagnosis of IBS using box plots and classical univariate ROC curve analyses. Quinate, also known as quinic acid, was recently reported that can promote the survival of endometriotic epithelial cells in vitro and lesion growth in vivo, suggesting the disease-promoting potential of microbiota-derived metabolites [24]. However, quinate has not been reported in BS. SDA is one of Omega-3 polyunsaturated fatty acids. Several recent studies have explored its potential health benefits and preventive roles in inflammation, cardiovascular disease (CVD) and cancer [25]. In this study, we found the level of SDA to be significantly higher in IBS patients than that in the NC group. Recently, Park SJ found that polyunsaturated fatty acids including fatty acids (FA) 20:4;2O, oxidized arachidonic acid, FA 20:4;3O, hypoxanthine, sphingosine 1-phosphate (S1P), and FA 18:2 (linolenic acid) were signifcantly increased in BS patients compared to that in healthy individuals [9], which was consistent with our above finding. CA (C10:0), a small molecule, performs important therapeutic implications for treating many diseases. A previous study confirmed that CA activates calcitriol sensitization in colon cancer cells and could be used as a successful supplement for intestinal diseases and colon aberrations [26]. In this study, we found CA is a potential biomarker for diagnosis of IBS. On the other hand, m1A, genipin, MMA and ascorbate were found to be the potential biomarkers for disease activity assessment of IBS. Additionally, m1A, genipin, MMA and ascorbate were positively correlated with ESR, CRP and BDCAF. M1A (N1-methyladenosine or 1-methyladenosine), which is abundant in human mRNAs [27], is a unique type of base methylation in that it blocks Watson–Crick base pairing, introduces a positive charge and can dramatically alter protein-RNA interactions and RNA secondary structures through electrostatic effects [28]. M1A is responsive to many kinds of cellular stress [27, 29]. Wang YY et al. found m1A methylation in tRNA could drive liver tumourigenesis by regulating cholesterol metabolism [30]. Genipin is an aglycone derived from the geniposide, which has long been used in traditional oriental medicine for the prevention and treatment of several inflammation driven diseases [31]. Li et al. found genipin can attenuate dextran sulfate sodium-induced colitis by suppressing inflammatory and oxidative responses [32]. Methylmalonic acid (MMA), a new potential biomarker, has been widely reported to be associated with the progression and prognosis of chronic diseases such as cardiovascular events, renal insufficiency, cognitive impairment and cancer [33]. However, there have been few studies on m1A, genipin and MMA in BS. Ascorbate, also known as, vitamin C, is a cofactor for several α-ketoglutarate-dependent dioxygenases, which has many biological activities that involve fundamental cellular functions such as gene expression, differentiation, and redox homeostasis [34]. A previous study reported that ascorbate levels in serum were significantly lower in both active and inactive BS patients compared with the control group [35], which was inconsistent with the results in IBS patients of this study. Chambers JC et al. found ascorbate could improve the impaired vascular endothelial function in BS [36]. However, no studies have examined ascorbate levels specifically in IBS patients. A previous study reported diseased intestine from patients with Crohn's disease contained significantly more ascorbate than the adjacent macroscopically normal intestine [37]. The expression and role of ascorbate in intestinal injury of BS are still unclear. Therefore, further research is needed to explore the expression levels and function of ascorbate in patients with IBS.

There were several limitations about this study which should be noted. Firstly, our results may not be generalizable to other countries because most of the patients were Chinese who had relatively unique and homogenous genetic backgrounds. Secondly, selection bias was unavoidable because of the study design. Finally, our study was conducted in a single center with a relatively small size. Therefore, multi-center and larger samples are needed for validating the above biomarkers for diagnosing and predicting IBS in the future.

## Conclusion

In conclusion, this study demonstrated the characteristic plasma metabolic profiles between the IBS and NC groups, and between active and inactive IBS, using an untargeted LC/MS metabolomics profiling approach. In this study, quinate, SDA and CA were found to be potential biomarkers for diagnosis of IBS, and m1A, genipin, MMA and ascorbate were found to be potential biomarkers for disease activity assessment of IBS. These findings may provide insights for developing therapeutic strategies to manage IBS in the future.

## Supplementary Information

Supplementary material 1.

Supplementary material 2.

Supplementary material 3.

## Data Availability

Our data cannot be shared openly. Because we promised to protect the study participants’ privacy.

## Abbreviations

BS: Behçet’s syndrome
BDCAF: Behcet’s Disease Current Activity Form
CRP: C reactive protein
ESR: Erythrocyte sedimentation rate
SDA: Stearidonic acid
CA: Capric acid
m1A: 1-Methyladenosine
MMA: Methylmalonic acid
