# Supplementary material for: Specific plasma metabolite profile in intestinal Behçet’s syndrome
Source: Orphanet J Rare Dis. 2025 Jan 13;20:21. doi: 10.1186/s13023-024-03484-4 (PMC11727545; doi:10.1186/s13023-024-03484-4)
Supplement: Supplementary file 1 — Supplementary material 1. [file 13023_2024_3484_MOESM1_ESM.docx]

**A**


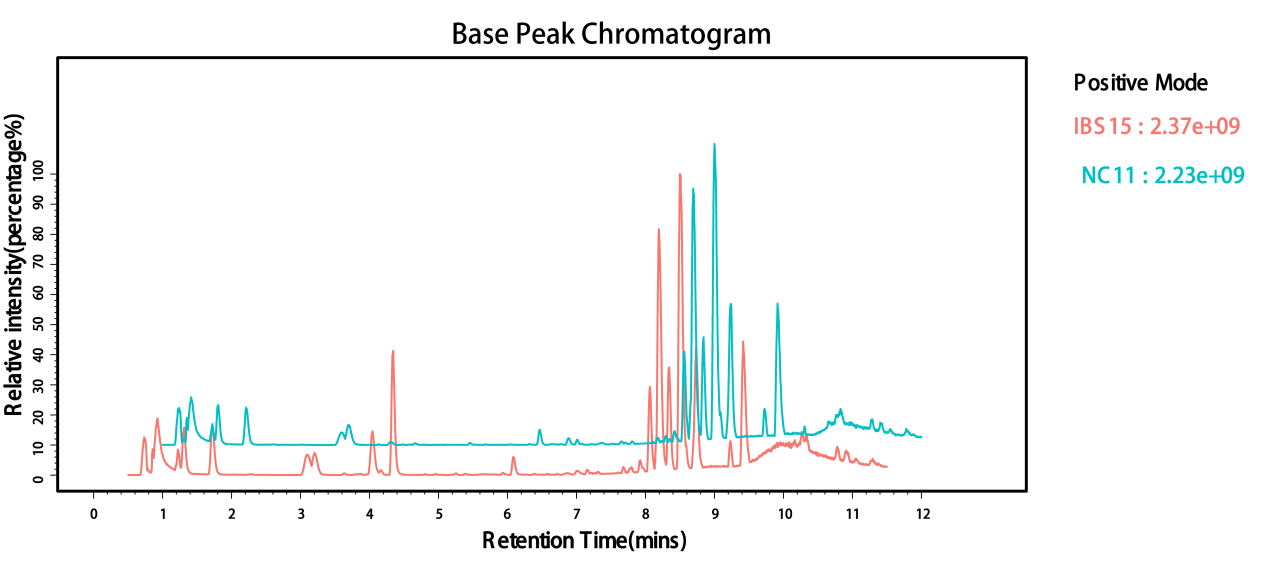


**B**

**
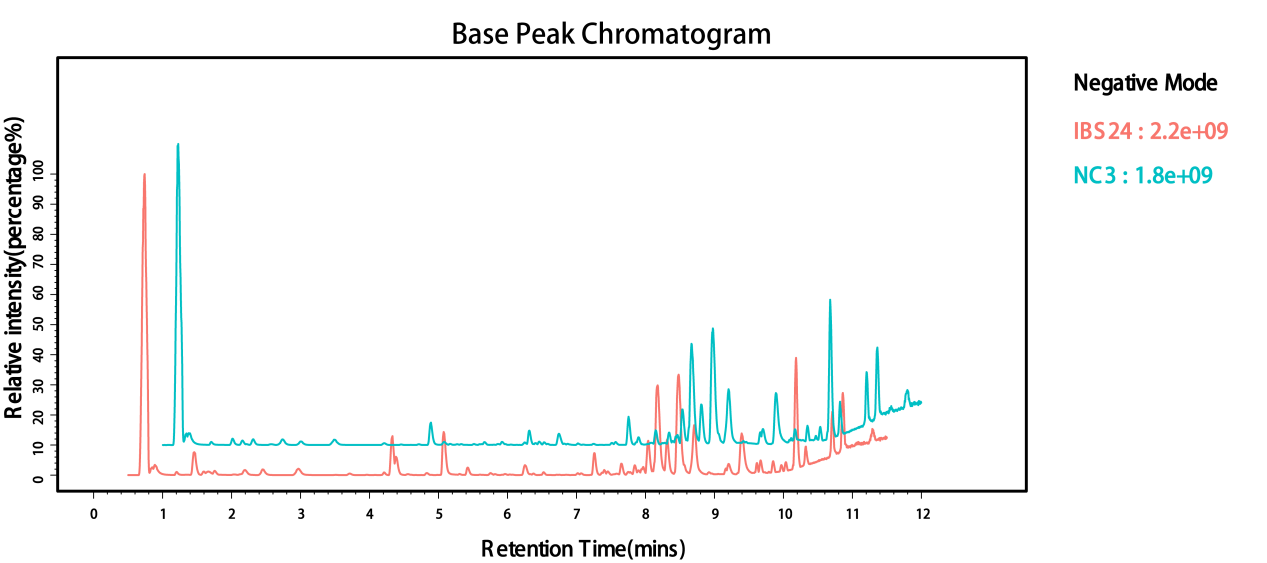
**

**C**


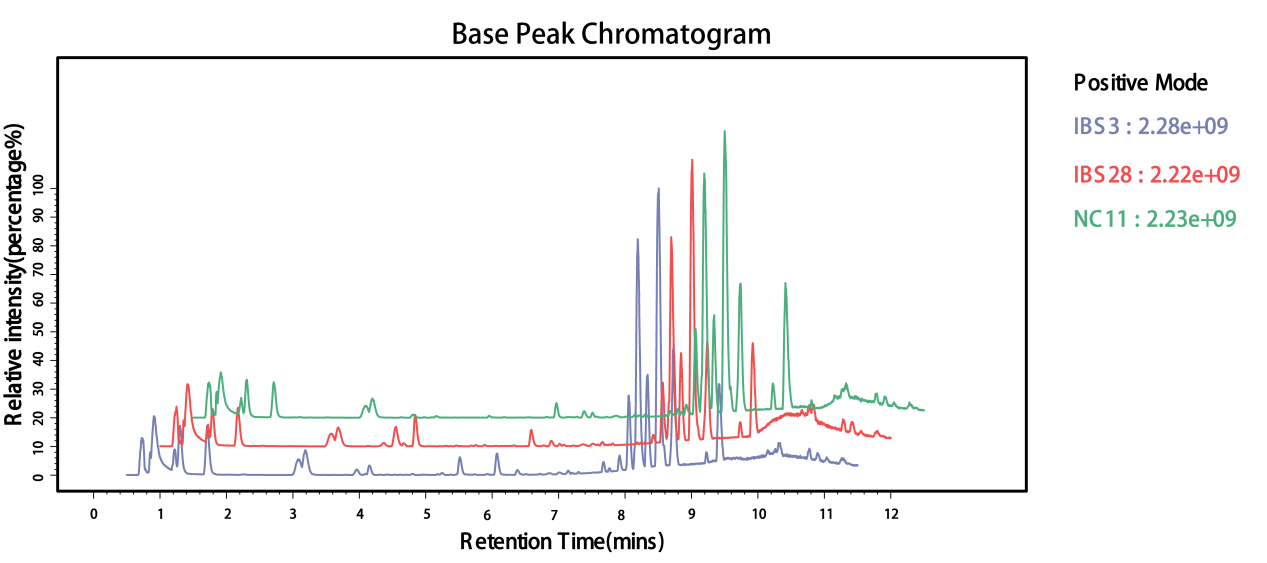


**D**

**
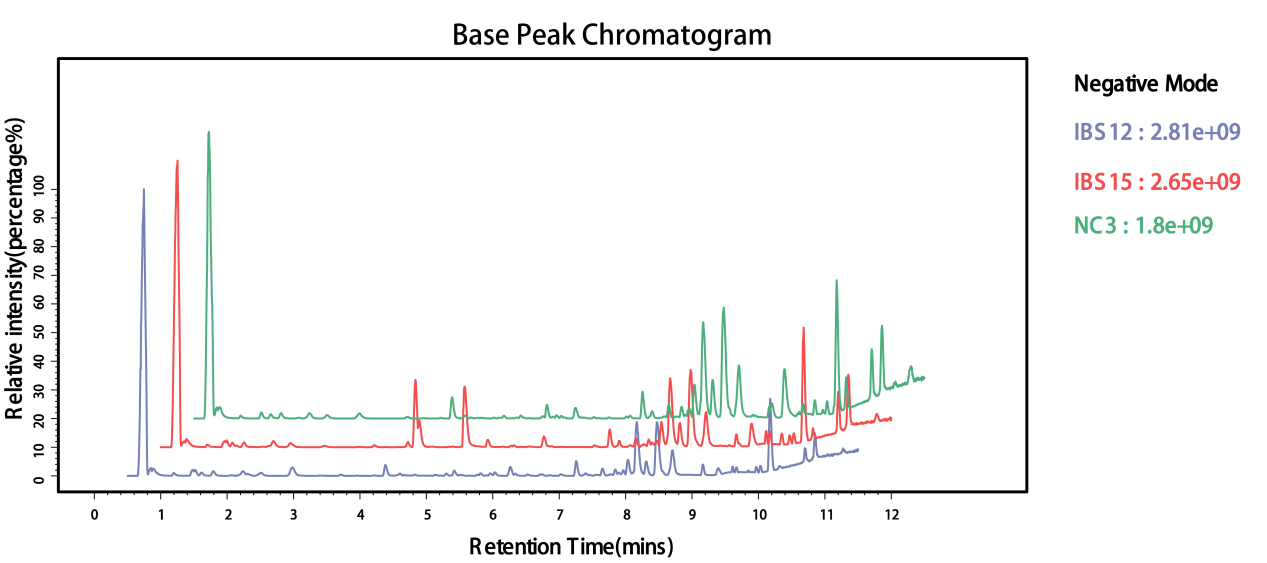
**

**Supplementary Figure 1. Representative LC-MS chromatograms of plasma**

**derived from IBS patients and NC.**

(A) The typically spectra (base peak chromatogram) of positive ion mode between IBS and NC. (B) The typically spectra (base peak chromatogram) of negative ion mode between IBS and NC. (C) The typically spectra (base peak chromatogram) of positive ion mode among active IBS, inactive IBS and NC. (D) The typically spectra (base peak chromatogram) of negative ion mode among active IBS, inactive IBS and NC.

**A**

**
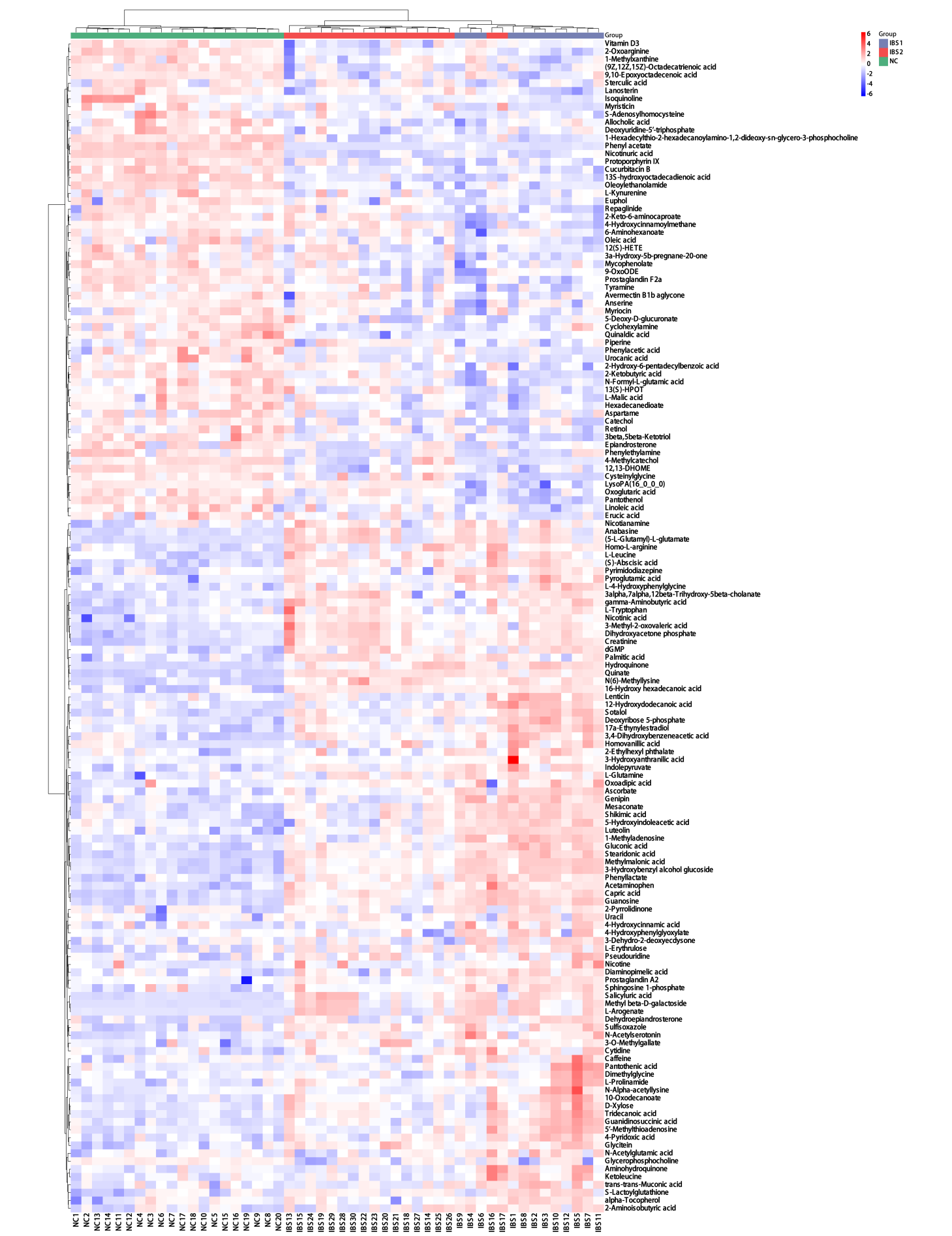
**

**B**

**
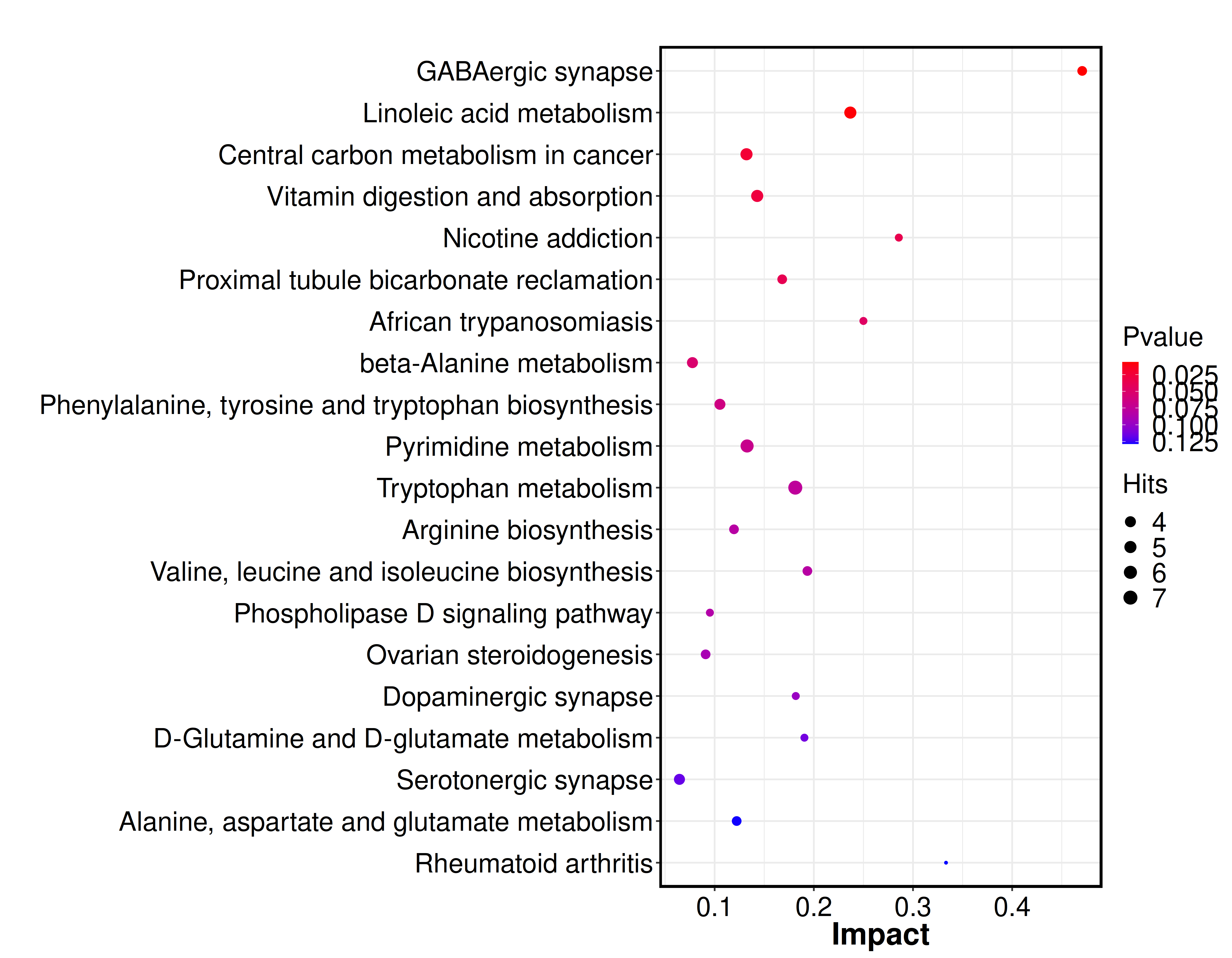
**

**Supplementary Figure 2. Differentially abundant metabolites and bubble diagram of KEGG pathway analysis among NC group, inactive IBS and active IBS patients.**

1. The hierarchical clustering heat map of the 149 metabolites. The rows represent the 149 metabolites, and the columns represent samples in NC group, inactive IBS and active IBS patients.
2. Bubble diagram of KEGG pathway analysis of differentiating metabolites enriched among NC group, inactive IBS and active IBS patients. The color of the bubbles represents the value of adjusted *P* value, and the size of bubbles represents the number of counts (sorted by enrichment ratio).

IBS 1: Active IBS patients; IBS 2: Inactive IBS patients; NC: normal contral.
